# Hepatitis E Virus Infection in a Liver Transplant Recipient Presenting With Late-Onset Acute Rejection and Treated With Ribavirin

**DOI:** 10.1155/crit/6565055

**Published:** 2025-09-23

**Authors:** Daffolyn Rachael Fels Elliott, Jwan Alallaf, Devin Shrock, Katie L. Dennis, Ryan M. Taylor, Maura F. O'Neil

**Affiliations:** ^1^Department of Pathology and Laboratory Medicine, University of Kansas Health System, Kansas City, Kansas, USA; ^2^Division of Gastroenterology/Hepatology, Department of Internal Medicine, University of Kansas Health System, Kansas City, Kansas, USA

**Keywords:** acute rejection, allograft hepatitis, hepatitis E virus, liver transplant

## Abstract

Hepatitis E virus (HEV) is increasingly recognized as a cause of allograft hepatitis in transplant recipients. Persistent HEV infection is often asymptomatic with nonspecific findings on liver biopsy, requiring high clinical awareness to order viral testing. We present a challenging case of HEV allograft hepatitis that presented with increasing liver enzymes and subtle biopsy findings of hepatocyte apoptosis and mitosis, diagnosed 1 month after treatment of acute rejection. Viral testing was positive for HEV IgM and HEV RNA, consistent with acute HEV infection. The patient was treated with a 12-week course of ribavirin. Based on this index case, we reviewed whether liver allograft biopsies with apoptosis were associated with viral detection at our institution during a 6-year period (*n* = 69) and compared histologic features of viral hepatitis and hepatitic patterns of rejection. Viral testing was performed in a majority (96%) of cases, but only five (7%) were tested for HEV. Anti-HEV IgG indicated prior exposure in one case. Clinicians should be aware there is histologic overlap between acute rejection and viral infection, including HEV, and etiologies may occasionally coexist. HEV testing should be considered in transplant recipients with unexplained elevated liver enzymes, particularly if biopsy demonstrates a hepatitic pattern or liver enzymes fail to normalize following treatment for rejection.

## 1. Introduction

Hepatitis E virus (HEV) is increasingly recognized as a cause of allograft hepatitis in liver transplant recipients. Transplant recipients can acquire HEV infection after ingesting undercooked meat, traveling to an endemic country, or by receiving infected human organs and/or blood transfusions. Additional risk factors in the liver transplant population include immunosuppression with calcineurin inhibitors, coinfection with hepatitis B virus (HBV) or human immunodeficiency virus (HIV), and recurrent chronic liver disease [1].

The clinical presentation of acute HEV infection is variable, ranging from mild subclinical hepatitis to jaundice and fulminant liver failure in patients on chronic immunosuppression, underlying chronic liver disease, and pregnant women [1]. Persistent HEV infection can develop in immunosuppressed patients who are unable to spontaneously clear the virus, defined by detectable HEV RNA for 3 months or longer [2, 3]. Persistent HEV infection is often asymptomatic with mild elevations in liver enzymes, requiring a high index of clinical suspicion to order viral testing. Liver biopsy findings are nonspecific and may be misdiagnosed as acute rejection or drug-induced liver injury [4]. A subset may progress to chronic hepatitis characterized by persistently elevated liver enzymes, detectable serum HEV RNA, and histologic findings of chronic hepatitis on liver biopsy [5, 6]. If untreated, chronic HEV infection can lead to liver fibrosis, cirrhosis, and potential allograft loss [5, 7].

Virus-specific testing for HEV RNA and/or antigen is recommended for the diagnosis of HEV infection in transplant recipients [3]. This is due to variability in antibody detection, since HEV seroconversion may be delayed or not occur, and antibody titers are unreliable in immunosuppressed patients [3, 8, 9]. Quantification of serum HEV RNA levels is important for diagnosis and to monitor response to therapy. Ribavirin monotherapy has been proposed as an effective agent to treat chronic HEV infection in liver transplant recipients [3, 8, 10, 11], although management guidelines for acute HEV infection are not well established. Other treatment strategies include reducing immunosuppression and minimizing calcineurin inhibitors if feasible.

Herein, we describe a case of acute HEV infection which presented with increasing liver enzymes after an episode of presumed late-onset T-cell–mediated rejection and was successfully treated with ribavirin monotherapy. Given the subtle biopsy findings of apoptosis and mitosis, we evaluated hepatitic patterns of rejection, viral detection, and HEV testing in a retrospective cohort of liver allograft biopsies at our institution.

## 2. Case Presentation

**Figure 1:**
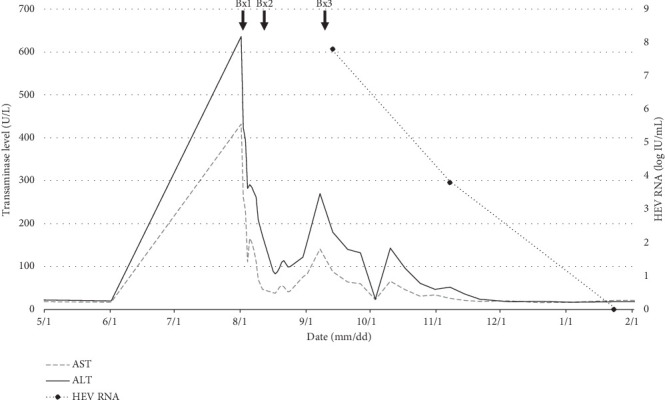
Timeline demonstrating the trend of liver transaminase (AST and ALT) elevation and serum HEV RNA levels for the index case with three consecutive liver biopsies (Bx).

A 71-year-old man with a history of liver transplantation 26 years ago for hepatitis C cirrhosis presented with elevation in liver enzymes at a routine follow-up visit. His early posttransplant course was complicated by an episode of acute rejection and recurrent hepatitis C treated with combination interferon and ribavirin therapy with sustained virologic response. At a routine follow-up visit, his liver enzymes were found to be elevated: AST 432 U/L, ALT 636 U/L, ALP 480 U/L, and total bilirubin 1.3 mg/dL (Figure 1). Tacrolimus level was within therapeutic range at 5.3 ng/mL. Doppler ultrasound of the liver showed patent hepatic vasculature. He underwent endoscopic retrograde cholangiopancreatography (ERCP), which showed no biliary stricture or anastomotic narrowing. An endoscopic ultrasound (EUS)-guided liver biopsy was performed, and a temporary biliary stent was placed.

**Figure 2:**
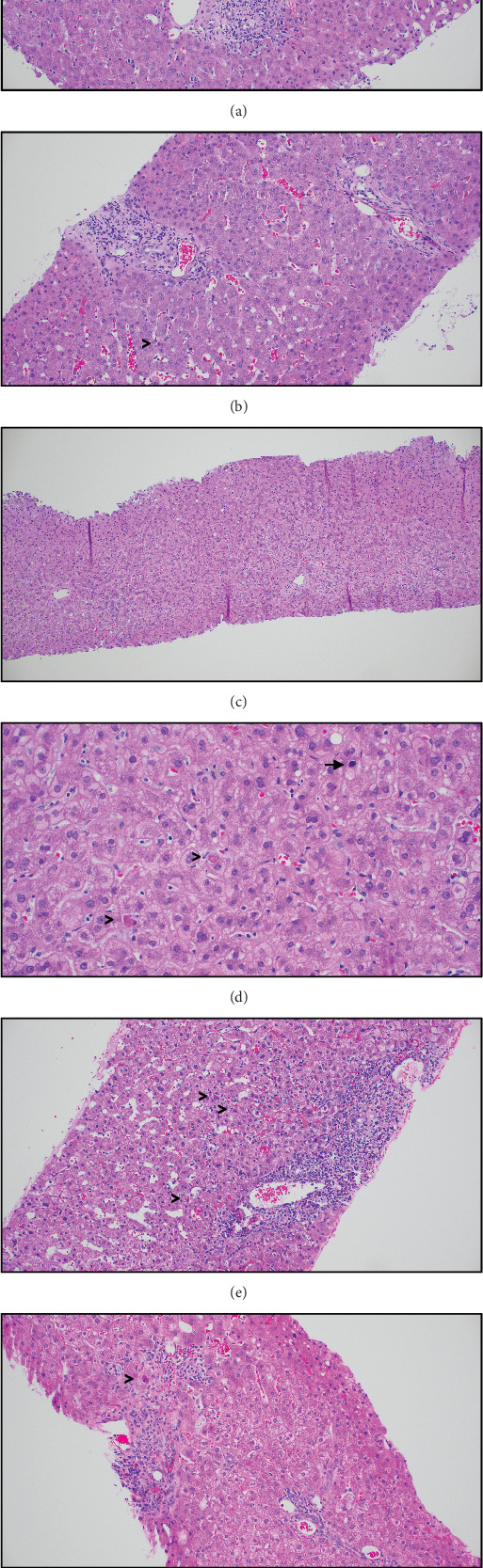
Sequential liver allograft biopsies for the index case of hepatitis E virus infection and overlapping histologic features with T-cell–mediated rejection. (a) The first biopsy (Bx1) at initial presentation with elevated liver enzymes shows mild portal lymphocytic inflammation with ductulitis and rare apoptosis (arrowhead), diagnosed as mild T-cell–mediated rejection (RAI 4/9). (b) The second biopsy (Bx2) 7 days after steroid therapy shows mild portal inflammation with bile duct damage and rare apoptosis (arrowhead), diagnosed as predominantly resolved T-cell–mediated rejection (RAI 1/9) (H&E, ×200). (c) The third biopsy (Bx3) 1 month later shows minimal portal inflammation (H&E, ×100). (d) At higher magnification, hepatocytes have a reactive appearance with increased mitosis (arrow), scattered apoptosis (arrowheads), and pigmented macrophages (H&E, ×400). (e) Similar histologic features in a case of moderate T-cell–mediated rejection (RAI 5/9) with scattered apoptosis (arrowheads) and (f) a case of resolving mild T-cell–mediated rejection (RAI 3/9) with rare apoptosis (arrowhead) (H&E, ×200).

Histologic sections of the EUS-guided liver biopsy demonstrated mild portal lymphocytic inflammation with minimal interface activity, ductulitis, and focal endotheliitis of portal venules (Figure 2a). A diagnosis of mild T-cell–mediated rejection was rendered, with Rejection Activity Index (RAI) of 4/9. He was treated with intravenous (IV) methylprednisolone 500 mg daily for 3 days. He developed post-ERCP pancreatitis requiring hospitalization for 1 week. Follow-up percutaneous liver biopsy performed 1 week after the initial biopsy showed predominantly resolved rejection (RAI 1/9) with rare apoptosis (Figure 2b). Liver enzymes were trending down: AST 47 U/L, ALT 172 U/L, ALP 275 U/L, and total bilirubin 1.4 mg/dL.

He was discharged from the hospital on an oral prednisone taper (starting at 15 mg, tapering by 2.5 mg every other week), and maintained on tacrolimus 3.5 mg twice daily with a target level of 8 ng/mL. He underwent follow-up ERCP with biliary stent removal. Despite initial down-trending liver tests, during the next 2 weeks, his liver enzymes increased, prompting a third liver biopsy. Laboratory values were AST 141 U/L, ALT 270 U/L, ALP 368 U/L, total bilirubin 1.1 mg/dL, and tacrolimus level 8.4 ng/mL. The repeat liver biopsy showed scattered apoptosis with small clusters of pigmented macrophages in the lobules and increased hepatocyte mitoses (Figure 2c,d). These histologic findings raised concern for an infectious etiology or possibly hepatic arterial insufficiency. Cytomegalovirus (CMV) immunohistochemistry and Epstein–Barr virus (EBV) *in situ* hybridization were negative for viral inclusions. Magnetic resonance angiography (MRA) of the abdomen showed patent hepatic vasculature.

Extended viral testing revealed positive HEV IgM and detectable HEV RNA at 56,600,000 IU/mL in serum by quantitative reverse transcription PCR (RT-qPCR), consistent with acute HEV infection. Additional viral tests for HAV, HBV, HCV, CMV, and EBV were negative. He was treated with a 12-week course of ribavirin 200 mg daily alternating with 400 mg daily due to underlying renal dysfunction. During follow-up, there was an on-treatment response with a decreased HEV RNA level to 5910 IU/mL at 8 weeks. HEV RNA was not detectable following the completion of ribavirin therapy and remained negative 5 months after treatment.

## 3. Results

### 3.1. Retrospective Review

To further investigate the association between liver biopsy findings of apoptosis and viral testing at our institution, we retrospectively reviewed liver allograft biopsies from 2016 to 2022 using database search terms *apoptosis*, *apoptoses*, *apoptotic*, *acidophil*, and *spotty necrosis.* We compared histologic features of hepatitic patterns of rejection and viral hepatitis and reviewed laboratory data including liver transaminases and viral testing. The database search identified 69 allograft biopsies from 58 liver transplant patients (34% female, median age 59.5 years) with histologic findings of apoptosis, including 36 acute/resolving rejection and 4 indeterminate for rejection (Table 1). Laboratory viral testing by serology and/or serum quantitative PCR was performed in 66 (96%) cases, including 5 (7%) tested for HEV, 60 (87%) for CMV, 12 (17%) for EBV, 13 (19%) for HBV, and 25 (36%) for HCV. Then, 18 (26%) cases were consistent with viral infection (10 HCV, 1 HBV, 5 CMV, 2 EBV), and 12 (17%) had low-level CMV viremia of uncertain significance. Positive anti-HEV IgG indicated prior exposure in one case.

Of the cases with acute/resolving rejection and apoptosis, 58% (21/36) were negative for infection. These included 12 cases of acute/resolving T-cell–mediated rejection with histologic features similar to the index case (Figure 2e,f). Additional patterns of rejection with apoptosis included plasma cell–rich hepatitis (5), isolated central perivenulitis (3), and chronic ductopenic rejection with central perivenulitis (1). A subset showed concurrent apoptosis and mitosis (6, none with arterial abnormality by imaging), sinusoidal lymphocytosis (2), and neutrophilic microabscesses (2). Persistent/resolving rejection had lower ALT compared to acute rejection (median 95 vs. 205, *p* = 0.01, Mann–Whitney test), and hepatocyte cytoplasmic clearing and ceroid-laden macrophages were prominent. Central perivenulitis favored rejection (52%, 11/21) but was also noted in 17% (3/18) of viral hepatitis cases (two with resolving rejection). Granulomas suggested infection in 17% (3/18) of viral hepatitis cases and were not observed in rejection.

## 4. Discussion

This case report illustrates the diagnostic challenge of HEV infection in the liver transplant population presenting with elevated liver enzymes and nonspecific biopsy findings. Classic clinical, laboratory, and histologic findings such as cholestatic hepatitis may be absent in the acute and chronic phases of HEV infection [5]. Our index patient was incidentally found to have elevated liver enzymes on routine laboratory tests and diagnosed with late-onset T-cell–mediated rejection based on the initial liver biopsy. His initial response to methylprednisolone seemed in keeping with rejection, with down-trending liver enzymes and features of resolving injury on follow-up biopsy. However, an alternative diagnosis was suspected after a third biopsy showed a mild hepatitic pattern, raising suspicion for viral infection.

The concurrent increase in apoptosis and mitosis also raised the possibility of vascular ischemia (e.g., secondary to hepatic artery thrombosis/stenosis) [12], which was excluded by imaging. The patient did not have any drug exposures and was adherent to immunosuppressive medications. In hindsight, we considered whether the patient may have had undetected HEV infection given the delay in viral testing, and we cannot exclude HEV coexisting with acute rejection. Extrahepatic manifestations of HEV infection have been described in transplant recipients, including acute pancreatitis [9, 13]. Interestingly, this patient developed post-ERCP pancreatitis, and it is uncertain whether this was related to acute HEV infection.

Diagnosis of HEV infection is challenging and requires a high index of clinical suspicion because histologic findings can be nonspecific, and liver biopsy may not reliably distinguish between acute rejection and viral infection [14]. Lenggenhager et al. described a broad spectrum of histologic findings of hepatitis E in liver samples from a large European cohort (41 patients) enriched for HEV Genotype 3 [4]. The authors observed that subclinical, smoldering hepatitis occurred more frequently in immunosuppressed patients, including a pattern of minimally active hepatitis similar to our index case. HEV genotyping is not routinely performed at our institution, which is a limitation, although nontravel-related HEV infections in the United States are predominantly Genotype 3 [15].

A few similar cases of hepatitis E mimicking liver allograft rejection have been reported in the literature with different approaches to viral testing and treatment (Table 2) [16–19]. Yoo et al. described a challenging diagnosis of hepatitis E in the United States due to variability in serologic assays, ultimately leading to graft fibrosis and death despite the initiation of ribavirin [16]. Thorburn et al. documented nearly a 3-year delay in the diagnosis of chronic hepatitis E due to overlapping histologic features with acute rejection and lack of HEV testing in Australia. The patient was treated with ribavirin 1000 mg daily for 3 months with viral clearance [19]. In contrast, two European case reports obtained earlier HEV test results, although both patients were initially diagnosed with acute rejection following liver biopsy and started on IV methylprednisolone. One patient with a high serum HEV RNA titer was treated with ribavirin 1200 mg daily for 3 months with viral clearance [17], while another patient with a low viral titer spontaneously cleared the infection [18].

The prevalence of HEV infection is rising in the United States, where zoonotic transmission of HEV Genotype 3 originates from animal reservoirs [15, 20]. Despite this, the rate of acquired new infections in the general population is quite low in the United States, estimated at seven infections per 1000 susceptible persons per year [15]. An international meta-analysis by Hansrivijit et al. found HEV infection was relatively common in liver transplant recipients worldwide, with an estimated pooled prevalence of 27% by detection of HEV IgM, IgG, or RNA [1]. Similarly, Frankal et al. showed 11% of liver transplant recipients acquired laboratory evidence of HEV infection during their study period in a Swedish population [21]. Although a majority (96%) of cases in our database review had viral testing for more common viruses (e.g., CMV, EBV, HBV, and HCV), HEV testing was only ordered in 7%, and a single case of prior HEV infection was detected by positive IgG. We could be missing some cases of HEV infection due to a combination of undertesting and the lower sensitivity of serological assays in this population. European Clinical Practice Guidelines recommend HEV testing in all immunosuppressed patients with unexplained elevation in liver enzymes [8], and moving forward at our institution, we are ordering serum HEV RNA RT-qPCR (a send-out test) more frequently.

Our retrospective review of liver allograft biopsies demonstrated that HEV testing was infrequently performed at our institution, despite reporting histologic findings of apoptosis that can be associated with viral infection. Notably, there was histologic overlap between rejection and viral infection in these biopsies, including neutrophilic microabscesses, sinusoidal lymphocytosis, and concurrent apoptosis and mitosis, which were nonspecific features in our cohort. Central perivenulitis favored rejection but was also present in a minority (17%) of viral hepatitis cases, including two cases recently treated for rejection, suggesting that occasionally etiologies may overlap. Granulomas were associated with viral infection in a subset (17%) and were not seen in rejection in our cohort.

In summary, clinicians and pathologists evaluating liver allograft biopsies should be aware that the histologic findings in HEV infection may be indistinguishable from T-cell–mediated rejection, including atypical hepatitic patterns of rejection. Virus-specific testing for HEV should be considered in liver transplant patients with unexplained elevated liver enzymes, particularly if liver biopsy demonstrates a hepatitic pattern of injury, including subtle features such as increased apoptosis and mitosis, or if liver enzymes fail to normalize following treatment for rejection.

## Figures and Tables

**Table 1:** Viral detection and histologic findings in liver allograft biopsies with apoptosis.

**Histologic diagnosis**	**Cases ** **N** = 69	**Viral infection**	**Viral detection method**	**Histologic findings in liver allograft biopsies with apoptosis**					
				**Resolving injury pattern**	**Central perivenulitis**	**Sinusoidal lymphocytosis**	**Granulomas**	**Neutrophilic microabscesses**	**Concurrent apoptosis + mitosis**
Acute rejection	27			5	9	6	1	2	7

	2	CMV	Serum PCR	0	0	1	0	0	0
	1	EBV, CMV	Serum PCR, tissue EBER ISH	0	0	1	1	0	0
	6	Low-level CMV DNA	Serum PCR	3	0	2	0	0	2
	18	None		2	9	2	0	2	5

Resolving rejection	9			6	5	2	1	0	2

	2	HCV	Serum PCR	1	0	1	0	0	0
	1	HCV, CMV	Serum PCR	1	1	0	0	0	0
	1	EBV, low-level CMV DNA	Serum PCR	0	0	1	1	0	0
	1	CMV	Serum PCR	1	1	0	0	0	0
	1	Low-level CMV DNA	Serum PCR	1	1	0	0	0	1
	3	None		2	2	0	0	0	1

Indeterminate for rejection	4			3	0	0	0	0	1

	1	HCV	Serum PCR	0	0	0	0	0	0
	3	None		3	0	0	0	0	1

No rejection	29			9	1	2	1	3	8

	5	HCV	Serum PCR	1	1	0	0	0	1
	1	HCV, CMV	Serum PCR	0	0	0	0	0	0
	1	HBV, CMV	Serum PCR	0	0	0	0	0	1
	2	CMV	Serum PCR	2	0	0	1	0	0
	5	Low-level CMV DNA	Serum PCR	1	0	0	0	1	0
	15	None		5	0	2	0	2	6

**Table 2:** Case reports of hepatitis E virus (HEV) mimicking acute graft rejection on liver biopsy.

	**Yoo et al. ** **2013 [16]**	**Allaire et al. ** **2018 ** **[17]**	**Hillebrandt et al. ** **2018 [18** **]**	**Thorburn et al. ** **2024 [19** **]**
Age/sex	58 male	58 male	56 male	41 male
Country	United States	France	Germany	Australia
Time from transplant to HEV infection	5 years	7 months	17 years	5 months
Indication for biopsy	Elevated liver enzymes	Elevated liver enzymes	Elevated liver enzymes	Elevated liver enzymes
Biopsy #1	Moderate TCMR	Moderate TCMR	Mild TCMR	Indeterminate for rejection
Biopsy #2	Mild TCMR			Moderate TCMR
Biopsy #3	Viral hepatitis vs. DILI			Atypical rejection vs. viral hepatitis
Biopsy #4	Marked duct damage, periportal fibrosis			Atypical rejection vs. viral hepatitis
Delay in diagnosis	3 months (diagnosis made after third biopsy)	No	No	2 years 10 months (diagnosis made after fourth biopsy)
HEV serology	IgM +	IgM +	IgM +	IgG +
	IgG -	IgG +	IgG +	
Virus-specific testing (RT-qPCR)	Serum and stool HEV RNA	Serum HEV RNA (high viral load: 2,300,000 IU/mL)	Serum HEV RNA (low viral load)	Serum HEV RNA (high viral load: 497,644,925 IU/mL)
HEV genotype	3	3	Not tested	3
Treatment for acute rejection	IVMP, increased maintenance IS	IVMP 500 mg x1 day (stopped after HEV detected)	IVMP 500 mg x5 days, increased maintenance IS	Multiple rounds of IS, including IVMP and ATG
Treatment for HEV	Ribavirin initiated after fourth biopsy	Ribavirin 1200 mg daily for 3 months	None	Ribavirin 1000 mg daily for 3 months
Outcome	Death	Cure, viral load not detected	Spontaneous resolution	Cure, viral load not detected

Abbreviations: ATG, antithymocyte globulin; DILI, drug-induced liver injury; IS, immunosuppression; IVMP, intravenous methylprednisolone; RT-qPCR, quantitative reverse transcription polymerase chain reaction; TCMR, T-cell–mediated rejection.

## Data Availability

The datasets generated and analyzed during the current study are available from the corresponding author upon reasonable request.
